# On variations in micrographic surgery and the use of horizontal histological sections in the evaluation of the surgical margin – Reply^☆^

**DOI:** 10.1016/j.abd.2020.04.002

**Published:** 2020-05-17

**Authors:** Francisco Macedo Paschoal

**Affiliations:** Dermatology Sector, Faculdade de Medicina do ABC, Santo André, SP, Brazil

Dear Editor,

In their article, Portela et al. proposed a new way of evaluating the product of tumor enucleation using horizontal histological sections.^1^

Apart from the discussion on the different techniques of micrographic surgery, including the Munich technique,^2^ the similarity between the technique presented by Portela et al. and the Munich technique relates to the way the tissue is sectioned for histological analysis, *i.e.*, in horizontal or parallel sections to the skin surface. However, several differences can be listed. Enucleation or debulking is not necessarily performed through a vertical incision. Most of the time, the incision is tangential to the skin surface. The sections of the surgical specimen, as proposed by Portela et al., are made from the surface to the bottom, contrary to what is done in the Munich technique. This is justified, as the main objective of the histological evaluation by horizontal sections of the enucleated tumor is to allow a better analysis of the histological subtype and the tumor site. Therefore, it is more logical that the sections start on the surface, the level where the tumor is already present. The subsequent assessment of the surgical borders and margins in the study by Portela et al. was carried out as recommended in Mohs micrographic surgery.

It is worth mentioning that horizontal histological sections, also termed transverse sections, have been used for decades in dermatopathology, such as in hair follicle diseases and in the correlation between dermatoscopy, confocal reflectance microscopy, and histopathology.^3^, ^4^, ^5^

Therefore, the study did not aim to describe a new micrographic surgery technique, since Mohs micrographic surgery was used in the peripheral control of the margins. Nevertheless, the debate on the different types of micrographic surgery is of great importance, due to its growing diffusion and the progressive increase in the number of micrographic surgeons in Brazil.

## Financial support

None declared.

## Author's contributions

Francisco Macedo Paschoal: Approval of the final version of the manuscript; drafting and editing of the manuscript; critical review of the literature; critical review of the manuscript.

## Conflicts of interest

None declared.
